# Levels of the interleukins 17A, 22, and 23 and the S100 protein family in the gingival crevicular fluid of psoriatic patients with or without periodontitis^☆^^☆☆^

**DOI:** 10.1016/j.abd.2020.08.008

**Published:** 2021-01-25

**Authors:** Constanza Jiménez, Daniela Carvajal, Marcela Hernández, Fernando Valenzuela, Jessica Astorga, Alejandra Fernández

**Affiliations:** a Department of Oral Pathology, Faculty of Dentistry, Universidad Andrés Bello, Santiago, Chile; b Department of Dermatology, Faculty of Medicine, Universidad de Chile, Santiago, Chile; c Laboratory of Periodontal Biology, Faculty of Dentistry, Universidad de Chile, Santiago, Chile; d Department of Oral Pathology and Medicine, Faculty of Dentistry, Universidad de Chile, Santiago, Chile

**Keywords:** Cytokinesis, Periodontal diseases, Psoriasis, S100 proteins

## Abstract

**Background:**

Psoriasis and periodontitis are immunologically mediated chronic inflammatory diseases. Epidemiologic evidence has linked both; however, the change of markers in gingival crevicular fluid has been poorly evaluated.

**Objective:**

To evaluate the levels of IL-17A, IL-22, IL-23, S100A7, S100A8, and S100A9 in gingival crevicular fluid of psoriatic and healthy subjects with and without periodontitis and their relations to psoriasis severity.

**Methods:**

Cross-sectional study. Sample comprised the following groups: healthy controls without periodontitis or with mild periodontitis (n = 21), healthy controls with moderate or severe periodontitis (n = 18), individuals with psoriasis without or mild periodontitis (n = 11), and individuals with psoriasis and moderate or severe periodontitis (n = 32). Levels of IL-17A, IL-22, IL-23, S100A8, and S100A9 were determined by multiplex assay and S100A7 was measured by ELISA.

**Results:**

No inter-group differences in the levels of IL-17A, IL-22, IL-23, and S100A7 were found. S100A8 levels were higher in psoriatic patients than controls (p < 0.05). S100A8 was positively correlated with psoriasis severity in the group with psoriasis (p < 0.05). S100A9 exceeded the detection limits.

**Study limitations:**

This pilot study presents a small sample size.

**Conclusions:**

The concentrations of S100A8 were highest in psoriatic patients regardless of periodontal health/status. S100A8 was associated with the severity of psoriasis. The concentrations of interleukins and S100A7 were similar in psoriatic patients with or without periodontitis *vs.* healthy controls.

## Introduction

Psoriasis is a chronic immunologically mediated inflammatory dermatosis and recurrent autoimmune disease that occurs in genetically susceptible individuals. Psoriasis is common; its prevalence is about 2% in Europe and North America. The main clinical skin manifestations include itching, burning, and soreness, accompanied by the development of well-defined, scaly, erythematous plaque lesions resulting from abnormal proliferation and differentiation of keratinocytes.^1^

The pathogenesis of psoriasis remains unclear; however, interactions between keratinocytes and resident skin T cells within the interleukin (IL) 23/Th17 pathway appear to participate in the onset and progression of the disease.^2^ Evidence supporting this hypothesis includes the isolation of IL-23 and IL-17 mRNA from psoriatic skin lesions and the report of increased serum concentrations of IL-17A, IL-22, and IL-23 in psoriatic patients *vs.* healthy controls.^3^, ^4^, ^5^ In recent years, special attention has been given to the prospective role of S100 proteins in the development of psoriasis. The S100 protein family is composed of calcium-binding proteins, in which psoriasin (S100A7) and calgranulin A (S100A8) are regulated by IL-17, amplifying the inflammatory response. S100A7, S100A8, and calgranulin B (S100A9) have been reported to express in psoriatic skin-lesions revealing these S100 proteins could play a key role in the unregulated growth and differentiation of keratinocytes in the skin of psoriatic patients.^6^, ^7^, ^8^, ^9^ Additionally, it has been indicated S100 proteins act as a chemotactic factors for immune cells, inducing a pro-inflammatory environment in which the onset and recurrence of psoriasis may easily occur.^9^

Recently, epidemiological studies have associated psoriasis with periodontitis, suggesting a common underlying immunopathogenesis.^10^, ^11^ Although studies on the subject are scarce, results are particularly interesting since a potentially bidirectional relationship between the two diseases has been suggested. In these studies, psoriatic patients not only presented a higher frequency of periodontitis and worse periodontal parameters, but also an increased risk of developing periodontitis compared to non-psoriatic healthy controls.^12^, ^13^, ^14^ Furthermore, a potential dose-response relationship between psoriasis and periodontitis was reported by Egeberg et al.^10^ as the frequency of periodontitis increased when the severity of the psoriasis augmented. Some evidence indicates periodontal patients might have an increased frequency and risk of psoriasis; nonetheless, more evidence is still needed. In the same line as psoriasis, the pathogenesis of periodontitis includes a special involvement of the IL-23/Th17 pathway. The expression of IL-17, IL-22, and IL-23 has been evaluated in the gingival crevicular fluid (GCF) of patients with chronic periodontitis, and these cytokines have been associated with the pathogenesis and severity of the disease and alveolar bone resorption.^15^, ^16^, ^17^ In periodontitis, studies have detected a S100A8/S100A9 heterocomplex in GCF, saliva, and serum of patients with periodontitis, whereas a recent study associated periodontitis with elevated levels of salivary S100A8.^18^, ^19^, ^20^, ^21^ To date, there has been no evidence to evaluate the expression of IL-23/Th17-related cytokines and S100-family proteins in the GCF of psoriatic patients. Due to GCF comprising diagnosis biomarkers of oral and systemic diseases as well as accurately exposing the constituents of serum and the cellular reactions of the periodontium, it is hypothesized that the levels of cytokines involved in T-cell regulation and S100 protein family molecules in GCF will show marked differences in psoriatic and healthy individuals with or without periodontitis, reflecting the link between psoriasis and periodontitis.^22^ Hence, the aim of this study was to evaluate the levels of IL-17A, IL-22, IL-23, S100A7, S100A8, and S100A9 in GCF of psoriatic and healthy subjects with and without periodontitis and their relations to psoriasis severity.

## Methods

The present cross-sectional study was approved by the bioethics committee of the North Metropolitan Health Service, Santiago, Chile (N52/2017). All participants provided written informed consent prior to study enrollment, in accordance with the Helsinki Declaration.

### Selection of patients

Individuals referred for presumed diagnosis of psoriasis to the dermatology unit of San José Hospital, Santiago, Chile, were enrolled over a ten-month period (2018). Individuals without psoriasis were simultaneously selected from volunteering patients attending the dental clinic of the Faculty of Dentistry of Andres Bello University, Santiago, Chile. Global eligibility criteria included the following: I) adult patients (≥ 18 years) with II) eleven or more teeth (excluding third molars). Exclusion criteria were set as follows: I) individuals with any known systemic disorders/conditions other than psoriasis (especially those involving inflammatory/immune dysregulation such as diabetes, systemic lupus, and arthritis/osteoarthritis, among others), II) patients who had received antibiotic, nonsteroidal anti-inflammatory, and/or immunomodulatory therapy in the last three months, III) patients who had undergone radiotherapy and/or chemotherapy in the last year, and IV) patients who had received periodontal or/and psoriasis treatment in the last six months.

### Clinical measurements

All patients were evaluated by the same team of experts. Dermatological data were collected from the initial consultation by dermatologists from the hospital staff. Clinical confounder variables evaluated included sex and age. Clinical psoriasis variables included presence or absence of psoriasis, clinical type of psoriasis, and psoriasis severity, defined by the (a) Psoriasis Area and Severity Index (PASI), (b) body surface area (BSA), the (c) static Physician’s Global Assessment (PGA), and the Dermatology Life Quality Index (DLQI). Periodontal evaluation and sampling were performed by a qualified periodontist. Clinical periodontal evaluation included full-mouth manual periodontal charting (six sites per tooth, excluding third molars) and recording of the following parameters: probing depth (PD), clinical attachment level (CAL), bleeding on probing (BOP), and tooth loss. Additional information regarding oral hygiene habits and smoking were collected. Periodontal status was defined according to the clinical case definition of periodontitis proposed by the Centers for Disease Control and Prevention (CDC), United States. Severe periodontitis was defined as two or more interproximal sites with CAL ≥ 6 mm (not on the same tooth) and ≥ 1 interproximal site with PD ≥ 5 mm. Moderate periodontitis was defined as two or more interproximal sites with CAL ≥ 4 mm (not on the same tooth) and/or two or more interproximal sites with PD ≥ 5 mm. Finally, no/mild cases of periodontitis were defined as neither severe nor moderate periodontitis.^23^

From a total of 93 individuals evaluated, 82 met the inclusion criteria. Subjects were divided into the following groups: H, systemically healthy controls with no/mild periodontitis; P, systemically healthy controls with moderate/severe periodontitis; S, individuals with psoriasis with no/mild periodontitis; and SP, individuals with psoriasis and moderate/severe periodontitis. Finally, from an ethical standpoint, it must be noted that all patients examined in this study with a positive diagnosis for either psoriasis and/or periodontitis were referred for timely treatment at San José Hospital and/or the dental clinic of Andres Bello University, respectively.

### Gingival crevicular fluid (GCF) collection

Samples were obtained from four independent sites based on probing depth, deepest site per quadrant. Before sampling, sites were isolated with cotton rolls and then carefully dried with an air-syringe to prevent saliva contamination. GCF was collected using sterile periodontal strips (Periopaper®; Interstate Drug Exchange – Amityville, NY, United States) gently inserted into the gingival sulcus or pocket until mild resistance was noticed. After 30 seconds, strips were collected into sterile tubes and immediately transported to the laboratory for storage (−20 °C) and posterior analysis.

### GCF analysis

A 40 μL elution/strip was prepared using protein elution buffer into sterile tubes. Samples were incubated for half an hour at 4 °C and then centrifuged at 12,000 × g for five minutes at 4 °C. The procedure was repeated twice, and samples were frozen and kept at −20 °C until analysis. Aliquots from all GCF samples were used for protein quantification using a multiplex assay panel (Human Magnetic Luminex Assay®; R&D Systems – MN, United States) for Il-17A, Il-22, IL-23, S100A8, and S100A9, and an ELISA Kit (Human Protein S100-A7 Elisa Kit®; MyBioSource Inc – San Diego, CA, United States) for S100A7, according to the manufacturers’ instructions. Data from the multiplex analysis panel were read using a platform (Magpix; Millipore – St. Charles, MO, United States) and then analyzed using software (MILLIPLEX AnalystR; Viagene Tech – Carlisle, MA, United States).

### Statistical analysis

The Shapiro-Wilk test and Levene’s test were used to determine the distribution and homoscedasticity of the data, respectively. Inferential analyses were performed with Fisher’s exact test, analysis of variance (ANOVA), and Duncan’s post hoc test. An ANOVA model type III was applied with a covariate: the age of the subjects examined. The correlation coefficients were obtained using Spearman’s correlation analysis. The level of significance was defined as p *<* 0.05. The effect size (eta-squared) was calculated, shown in Table 1. The statistical analysis was performed using a statistical software (STATA 12®; StataCorp – College Station, TX, United States). It must be noted that as a result of the explorative nature of this research, no sample-size calculation was performed.

**Table 1:** Results of effect size for analysis of variance (ANOVA): eta squared.

Variables	Age (covariate)	Age (covariate)	Eta-squared groups
IL-17	F = 3.83 (p = 0.054)	F = 1.26 (p = 0.295)	0.047
IL-22	F = 1.19 (p = 0.279)	F = 0.36 (p = 0.784)	0.014
IL-23	F = 1.54 (p = 0.218)	F = 0.68 (p = 0.565)	0.026
S100A7	F = 0.23 (p = 0.629)	F = 0.57 (p = 0.635)	0.022
S100A8	F = 0.75 (p = 0.388)	F = 8.62 (p < 0.001)	0.251

## Results

In total, 82 individuals were included in this study: 21 controls with no/mild periodontitis (H), 18 controls with moderate/severe periodontitis (P), 11 individuals with psoriasis and no/mild periodontitis (S), and 32 individuals with psoriasis and moderate/severe periodontitis (SP).

Demographic data, habits, and clinical parameters of all individuals in this study are summarized in Table 2. No significant differences among groups regarding gender, smoking, BOP, and oral hygiene habits, PASI, BSA, PGA, and DLQI were found. The highest mean age was found in the SP group, followed by groups P, S, and H in decreasing order. Only the SP group showed significant differences in mean age with group H (p *<* 0.05).

**Table 2:** Comparison of demographic parameters, smoking habits, and clinical characteristics of study individuals.

Parameters	H	P	S	SP	p
Age (years, mean [SD])	33.52 (10.64)^a^	42.95 (12.48)	40.16 (16.82)	50.20 (11.43)^b^	**0.0001**
Gender: male-female (n)	9–12	12–6	5–6	13–19	0.342
Smokers (n, %)	6 (28.5%)	6 (33.3%)	3 (27.3%)	17 (53.1%)	0.235
PD (mm, mean [SD])	1.86 (0.24)^a^	2.24 (0.42)^b^	1.99 (0.5)^b,c^	2.53 (0.43)^d^	**0.0001**
CAL (mm, mean [SD])	1.33 (0.44)^b^	1.70 (0.64)^b^	1.56 (0.95)^b^	2.75 (1.26)^a^	**0.0001**
BOP (%, mean [SD])	19.36 (21.86)	12.80 (12.33)	10.16 (9.18)	11.60 (14.06)	0.240
Number of teeth (n, mean [SD])	25.24 (3.62)^b^	25(2.54)^b^	23.8(4.28)	21.67 (4.84)^a^	**0.003**
Frequency of dental brushing (n per day, mean [SD])	2.56 (0.76)	2.86 (0.88)	2.09(0.7)	2.24 (1.11)	0.054
PASI, mean (SD)	–	–	13.7 (7.91)	10.35 (6.40)	0.153
BSA, mean (SD)	–	–	19.20 (13.26)	16.08 (15.19)	0.531
PGA, mean (SD)	–	–	3 (0.6)	2.67 (0.76)	0.193
DLQI, mean (SD)	–	–	13.83 (6.8)	14.79 (7.71)	0.704

Bold, p < 0.05; H, healthy control; P, individuals with untreated periodontitis; S, individuals with untreated psoriasis; SP, individuals with untreated psoriasis and untreated periodontitis; SD, standard deviation; PD, probing depth; CAL, clinical attachment level; BOP, bleeding on probing; PASI, Psoriasis Area Severity Index; BSA, body surface area; PGA, Physician’s Global Assessment; DLQI, Dermatology Life Quality Index.

Superscript letters indicate significant differences among groups: ^a^ Present significant difference with whereas ^b^ whereas ^c^ present significant difference with^d^.

Regarding periodontal clinical parameters, PD was significantly worse/deeper in the SP group compared to groups S, P, and H (p *<* 0.05). The P group presented significantly deeper pockets than the H group (p *<* 0.05). The highest CAL was observed in the SP group compared to that for groups S, P, and H (p *<* 0.05). The highest tooth loss was observed in the SP group, followed by groups S, P, and H in decreasing order. Regarding dermatological clinical data, no significant differences in PASI, BSA, and PGA were found between groups S and SP.

**Figure 1:**
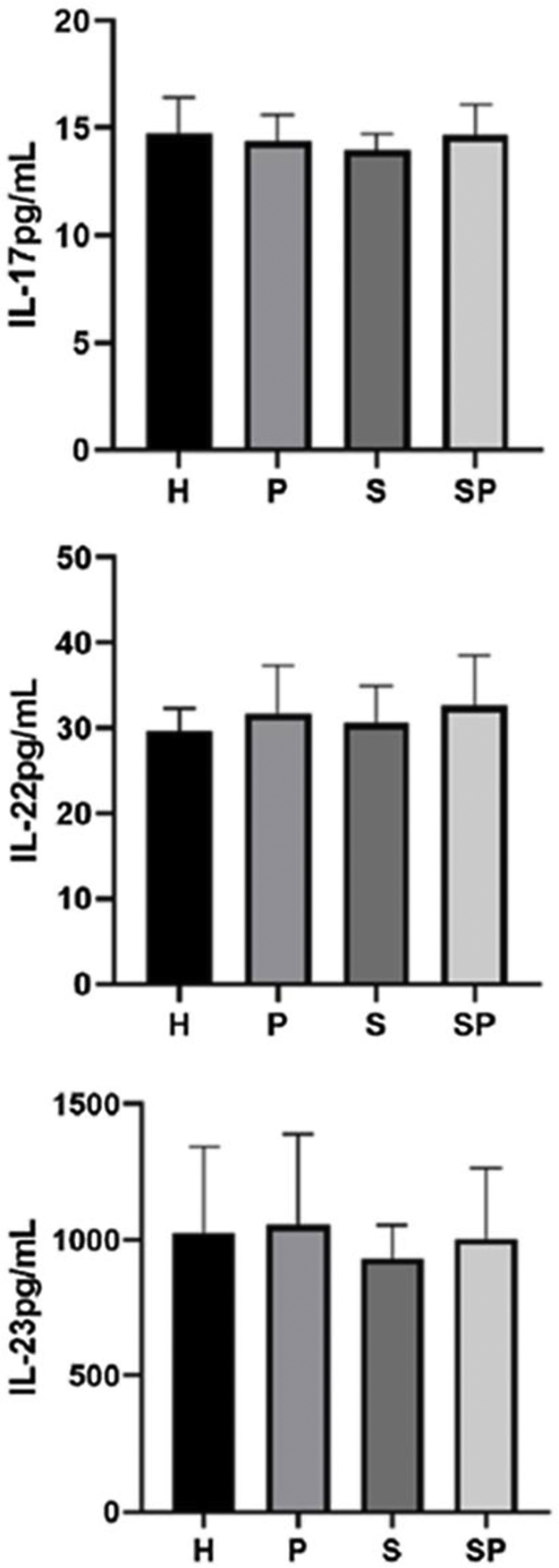
Concentration of IL-17, IL-22, and IL-23 in the GCF of study individuals. H, healthy control; P, individuals with untreated periodontitis; S, individuals with untreated psoriasis; SP, individuals with untreated psoriasis and untreated periodontitis.

**Figure 2:**
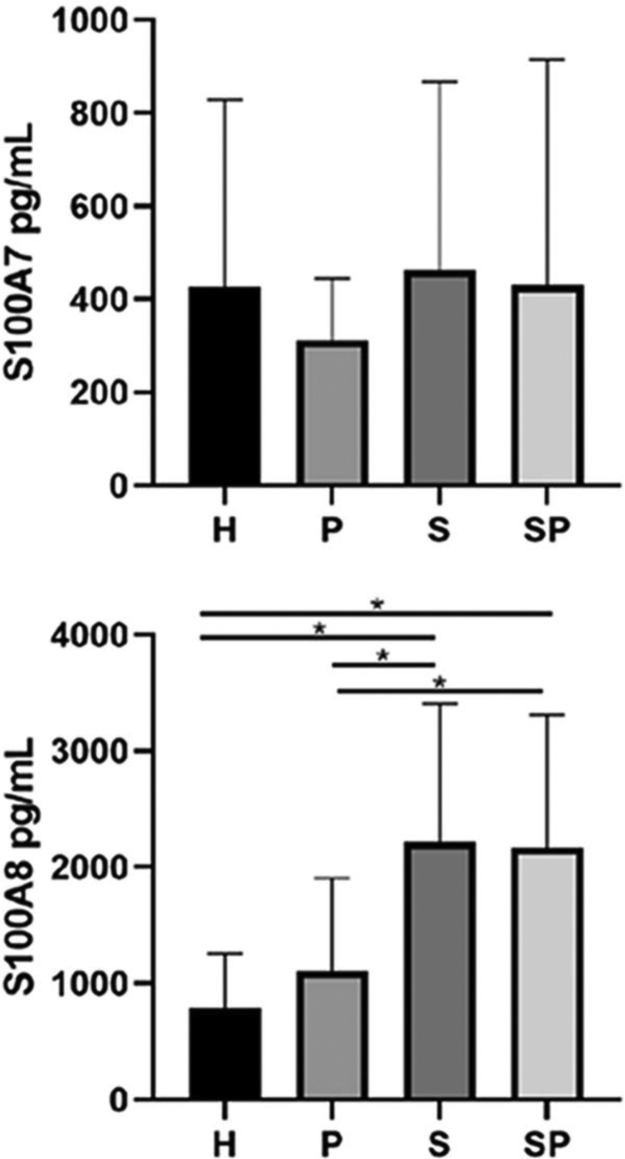
Concentration of S100A7 and S100A8 in GCF of study individuals. Bars and asterisk represent p.

The concentrations of IL-17A, IL-22, and IL-23 in the GCF are shown in Fig. 1. No intergroup differences regarding the levels of IL-17A, IL-22, and IL-23 in GCF were noted. The GCF levels of IL-17A, IL-22, and IL-23 in the SP group showed a tendency to be higher than those of the S group, respectively. GCF concentrations of S100A7 and S100A8 in the different groups are presented in Fig. 2. No significant differences in the levels of S100A7 in the GCF of the different groups were observed. Group H presented the lowest levels of S100A8 compared to the S and SP groups (p *<* 0.05). Additionally, group P presented lower levels of S100A8 compared to the S and SP groups (p *<* 0.05). Nevertheless, age demonstrated no influence upon the concentration of all interleukins and the S100 protein family (p > 0.05). Concentrations of S100A9 in the GCF of all groups exceeded the upper-detection limits of the ELISA kit; therefore, exact quantification and analysis were not possible.

The levels of inflammatory mediators in GCF were correlated with the periodontal clinical parameters in group H and group P. In group H, IL-17A levels were negatively correlated with CAL (-0.499), (p < 0.05). In group P, IL-22 was positively correlated with PD (0.490) and CAL (0.589) (p < 0.05). In groups S and SP, correlations between inflammatory mediators and both periodontal and dermatological clinical parameters were obtained. In the S group, IL-23 was negatively correlated with PGA, whereas S100A8 was positively and strongly correlated with PASI (0.878), BSA (0.851), and DLQI (0.622) (p < 0.05). In the SP group, IL-22 and IL-23 were negatively correlated with BSA (-0.358 and -0.468, respectively, p < 0.05).

## Discussion

Psoriasis and periodontitis share similar mechanisms of immune dysregulation, characterized by a Th17 axis-predominant immune response.^24^, ^25^ The Th17 pathway regulates the expression of S100 proteins, which may participate in the pathogenesis of both diseases.^26^, ^27^, ^28^ In the present study, elevated levels of S100A8 were found in the GCF of psoriatic patients compared to that of systemically healthy controls, regardless of their periodontal health/status; there was a strong positive correlation between S100A8 with BSA, PASI, and DLQI in the psoriasis group (group S), suggesting that GCF concentrations of S100A8 could be a promising non-invasive biomarker for the presence and severity of psoriasis.

To the best of the authors’ knowledge, no previous studies have evaluated the GCF concentrations of S100A7 and S100A8 in psoriatic individuals with or without periodontitis. In periodontitis alone, S100A7 and S100A8 have been detected in the GCF of individuals with periodontitis using the techniques of mass spectrometry and western blot, indicating these molecules might participate in the immune response of periodontal tissues.^29^ In addition, S100A7 and S100A8 could be involved in periodontal bone resorption, as evidence shows S100A8 induced osteoclastic bone resorption in experimental arthritis 30 and S100A7 stimulated osteoclast differentiation in human monocytes in vitro.^30^, ^31^ Recently, Karna et al. reported and associated increased levels of salivary S100A8 with periodontitis in Korean adults, suggesting salivary concentrations of S100A8 could be a prospective biomarker for periodontitis.^21^ In psoriasis, S100A7 has been reported to induce unregulated differentiation of the epidermis.^8^ It is thought that S100A7 functions as a keratinocyte-derived chemotactic agent for immune cells, as a result the molecule strongly attracts CD4+ lymphocytes and neutrophils under inflammatory stress. S100A8 and S100A9 molecules are initially released from keratinocytes to start/prompt immune cell invasion, which is further sustained by the later release of the same molecules from incoming neutrophils.^28^ The detection of different S100 family proteins in serum has been proposed as prospective biomarker for psoriasis, as increased levels of S100A7 and S100A8 have been detected in blood samples of untreated psoriatic patients compared with non-psoriatic healthy individuals. Moreover, gene expression of S100A7 and S100A8 in psoriatic skin lesions has been reported to be significantly higher than that of healthy skin samples.^28^

The present findings show S100A8 presents in higher concentrations in the GCF of psoriatic patients *vs.* non-psoriatic healthy controls, regardless of their periodontal health/status. Nonetheless, the concentrations of S100A7 between groups presented no differences. Therefore, in line with previously reported evidence, these results suggest that S100A8 may be a key protein involved in the pathogenesis of psoriasis. In addition, it is believed that S100A8 could reflect the presence and severity of psoriasis, thus presenting as a promising, safe, non-invasive biomarker for early diagnosis of the disease regardless of periodontal health. Increased levels of S100A8 in the GCF samples of psoriatic subjects might be explained by the systemic dissemination of the molecules from active psoriatic skin lesions: It is possible that active psoriatic skin lesions may function as local spots for the massive production of S100A8, which then enters the blood stream *via* increased vascular permeability and travels to distant sites, such as the oral gingiva.^32^

The present study observed that overall concentrations of IL-17A, IL-22, and IL-23 presented a tendency to increase in psoriatic patients with moderate/severe periodontitis (group SP) compared to psoriatic patients with no/mild periodontitis (group S). These findings could be attributed to a local effect of periodontitis. According to the literature, IL-17, IL-22, and IL-23 are associated with osteoclastogenesis in periodontitis; however, their concentrations within the oral fluids of periodontal patients remains controversial.^17^, ^33^, ^34^, ^35^ In one study, IL-17A levels in serum, saliva, and GCF were higher in periodontitis than in healthy controls.^36^ In contrast, another study reported higher levels of IL-17 in the GCF of healthy individuals than in subjects with either chronic or aggressive periodontitis.^37^ In relation to IL-22, a previous study reported no differences in the saliva concentrations of IL-22 of individuals with chronic periodontitis *vs.* healthy controls.^38^ However, an additional study reported higher levels of IL-22 in the GCF of subjects with periodontitis compared with those of subjects with gingivitis and healthy individuals 17. Regarding IL-23, a study reported higher concentrations of the molecule in the GCF of patients with chronic periodontitis *vs.* healthy controls.^39^ In contrast, R. Sadeghi et al. reported higher concentrations of IL-23 in the GCF of healthy subjects compared to patients with chronic periodontitis.^37^ It is theorized that these discrepancies may be due to differences in the methods used to quantify the concentrations of the above-mentioned proteins, as well as the absence of consensus regarding clinical case definitions of periodontitis.^23^

Previously, the detection of IL-17 in the serum and skin of patients with psoriasis has been reported, while not in GCF. Differences in the concentration of IL-17 in serum samples of psoriatic patients *vs*. those of healthy controls is controversial.^5^, ^40^, ^41^ However, skin psoriatic biopsies have shown higher expression of IL-17A than those of healthy subjects.^42^ Differences between the present results and those previously reported in the literature may be due to the different time points in the history of psoriasis.^5^ There are no previous studies reporting the presence of IL-17 in the GCF of individuals with psoriasis with or without periodontitis; however, higher levels of IL-17A have been detected in the GCF of individuals with other autoimmune diseases and periodontitis. Such is the case of rheumatoid arthritis patients with periodontitis, who have presented higher levels of IL-17A than those of systemically healthy subjects with periodontal disease.^43^ In addition, an experimental animal study has shown an increase in IL-17 production by lymph node T-cells in arthritic mice with periodontitis compared with that in arthritic mice without periodontitis.^44^ Another study showed that periodontitis seems to alter IL-17 levels in gingival tissue samples of arthritic rats.^45^

Regarding psoriasis, it has been reported that IL-22 and IL-23 are required to induce psoriasis-like lesions in animal models.^46^, ^47^ Similarly to the present results, some studies have reported no differences in the levels of IL-22 and IL-23 in the serum of patients with psoriasis *vs.* those of healthy controls.^48^, ^49^ However, only one study reported increased concentrations of IL-22 and IL-23 in the serum of psoriatic patients *vs.* those of non-psoriatic healthy controls.^5^ These findings suggest that IL-22 and IL-23 may act preferentially at a local level in psoriasis. All these antecedents support the potential role of periodontitis in the tendency to increased levels of IL-17, IL-22, and IL-23 in the SP group compared to the S group shown in the present study. In this study, the no/mild cases of periodontitis within groups H and S could have partially masked the net differences between healthy non-periodontal controls and periodontal patients.

Regarding the correlations between GCF inflammatory mediators and periodontal disease, it was found that group H had a negative correlation between the levels of IL-17A and CAL, whereas group P had a positive correlation between IL-22 and PD and CAL. These findings suggest that these inflammatory mediators participate in periodontal disease pathogenesis, which has been previously suggested in the literature, as both interleukins have been linked to osteoclastogenesis in periodontal disease.^17^, ^35^

Although the IL-23/Th17 pathway is part of cutaneous inflammation in psoriasis, a negative correlation was detected between IL-23 and PGA in group S. In addition, a negative correlation was found between IL-22 and IL-23 in group SP. This result could be explained by the diverging phenotype in psoriasis.^24^ To the authors’ knowledge, this study is the first article to report that GCF levels of S100A8 correlate positively with BSA, PASI, and DLQI in individuals with psoriasis, suggesting that S100A8 in GCF may serve as a potential biomarker of presence and severity of psoriasis. This may be justified by the high expression of S100A8 in the epidermis of psoriatic skin.^50^

This pilot study presents a small sample size, which the authors acknowledge as a limitation. Nonetheless, the number of individuals included allowed for the identification of changes in the GCF of individuals with and without psoriasis. Therefore, more studies are essential to elucidate the potential link between periodontitis and psoriasis.

## Conclusions

The concentrations of S100A8 were highest in the GCF of psoriatic patients regardless of their periodontal health/status. Furthermore, S100A8 in GCF was associated with the severity of psoriasis. The concentrations of interleukins and S100A7 were similar in GCF of psoriatic patients with or without periodontitis in relation to healthy controls. Therefore, the immune molecules evaluated in GCF in the present study do not seem to reflect the link between periodontitis and psoriasis.

## Financial support

This research was supported by a grant from Dirección General de Investigación de la Universidad Andrés Bello (DGI-UNAB), No. DI-36-18/CBC, and 10.13039/501100002850FONDECYT, No. 1160741.

## Authors' contributions

Constanza Jiménez: Approval of the final version of the manuscript; study conception and planning; preparation and writing of the manuscript; data collection, analysis, and interpretation; intellectual participation in propaedeutic and/or therapeutic conduct of studied cases; critical review of the literature; critical review of the manuscript.

Daniela Carvajal: Approval of the final version of the manuscript; preparation and writing of the manuscript; data collection, analysis, and interpretation; intellectual participation in propaedeutic and/or therapeutic conduct of studied cases; critical review of the literature; critical review of the manuscript.

Marcela Hernández: Approval of the final version of the manuscript; study conception and planning; preparation and writing of the manuscript; effective participation in research orientation; intellectual participation in propaedeutic and/or therapeutic conduct of studied cases; critical review of the literature; critical review of the manuscript.

Fernando Valenzuela: Approval of the final version of the manuscript; study conception and planning; effective participation in research orientation; intellectual participation in propaedeutic and/or therapeutic conduct of studied cases; critical review of the literature; critical review of the manuscript.

Jessica Astorga: Approval of the final version of the manuscript; collection, analysis, and interpretation of data; critical review of the manuscript.

Alejandra Fernández: Approval of the final version of the manuscript; study conception and planning; preparation and writing of the manuscript; data collection, analysis, and interpretation; effective participation in research orientation; intellectual participation in propaedeutic and/or therapeutic conduct of studied cases; critical review of the literature; critical review of the manuscript.

## Conflicts of interest

None declared.
